# Enhancing cell adhesive and antibacterial activities of glass-fibre-reinforced polyetherketoneketone through Mg and Ag PIII

**DOI:** 10.1093/rb/rbad066

**Published:** 2023-07-12

**Authors:** Xin Tan, Zhongyi Wang, Xin Yang, Ping Yu, Manlin Sun, Yuwei Zhao, Haiyang Yu

**Affiliations:** State Key Laboratory of Oral Diseases, National Clinical Research Center for Oral Diseases, West China Hospital of Stomatology, Sichuan University, Chengdu, China; Chongqing Key Laboratory of Oral Diseases and Biomedical Sciences, College of Stomatology, Chongqing Medical University, Chongqing, China; State Key Laboratory of Oral Diseases, National Clinical Research Center for Oral Diseases, West China Hospital of Stomatology, Sichuan University, Chengdu, China; Jiangsu Key Laboratory of Oral Diseases, Affiliated Hospital of Stomatology, Nanjing Medical University, Nanjing, China; State Key Laboratory of Oral Diseases, National Clinical Research Center for Oral Diseases, West China Hospital of Stomatology, Sichuan University, Chengdu, China; Department of Stomatology, Chengdu Second People’s Hospital, Chengdu, China; State Key Laboratory of Oral Diseases, National Clinical Research Center for Oral Diseases, West China Hospital of Stomatology, Sichuan University, Chengdu, China; State Key Laboratory of Oral Diseases, National Clinical Research Center for Oral Diseases, West China Hospital of Stomatology, Sichuan University, Chengdu, China; State Key Laboratory of Oral Diseases, National Clinical Research Center for Oral Diseases, West China Hospital of Stomatology, Sichuan University, Chengdu, China

**Keywords:** glass-fibre-reinforced polyetherketoneketone, plasma immersion ion implantation, human gingival fibroblasts, antibacterial activity

## Abstract

Glass-fibre-reinforced polyetherketoneketone (PEKK-GF) shows great potential for application as a dental implant restoration material; however, its surface bioinertness and poor antibacterial properties limit its integration with peri-implant soft tissue, which is critical in the long-term success of implant restoration. Herein, functional magnesium (Mg) and silver (Ag) ions were introduced into PEKK-GF by plasma immersion ion implantation (PIII). Surface characterization confirmed that the surface morphology of PEKK-GF was not visibly affected by PIII treatment. Further tests revealed that PIII changed the wettability and electrochemical environment of the PEKK-GF surface and enabled the release of Mg^2+^ and Ag^+^ modulated by Giavanni effect. *In vitro* experiments showed that Mg/Ag PIII-treated PEKK-GF promoted the proliferation and adhesion of human gingival fibroblasts and upregulated the expression of adhesion-related genes and proteins. In addition, the treated samples inhibited the metabolic viability and adhesion of *Streptococcus mutans* and *Porphyromonas gingivalis* on their surfaces, distorting bacterial morphology. Mg/Ag PIII surface treatment improved the soft tissue integration and antibacterial activities of PEKK-GF, which will further support and broaden its adoption in dentistry.

## Graphical abstract

**Figure:**
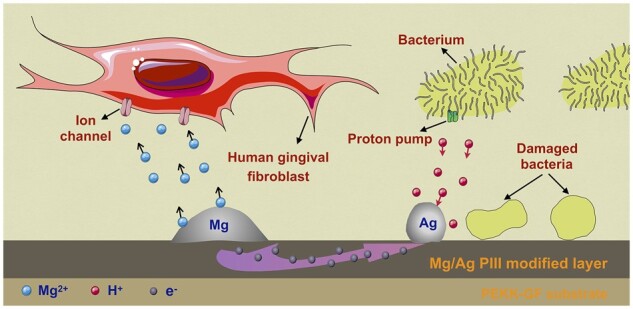

## Introduction

**Figure 1.**
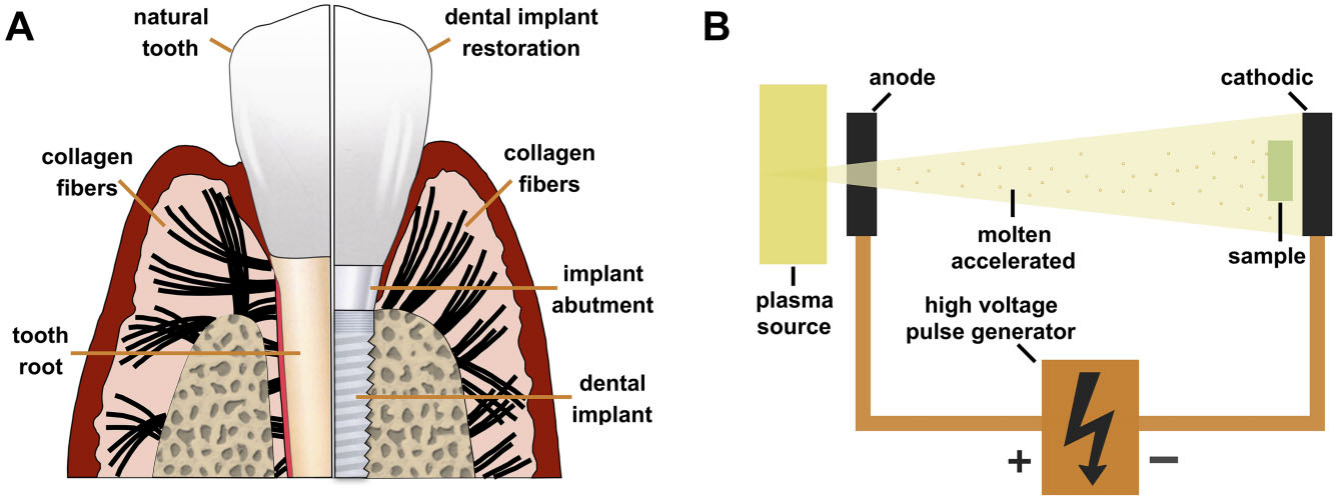
(**A**) Orientation of periodontal fibres in soft tissue around natural tooth and dental implant, which are radially located around tooth, while are parallel to the material surface around implant and are vulnerable to mechanical or microbial destruction. (**B**) Schematic illustration of the PIII treatment.

The long-term success and stability of dental implant restoration requires not only good osseointegration, but also ideal integration of implant materials with soft tissues in critical parts [1, 2]. For example, as a key structure connecting the dental implant and upper restoration, the implant abutment should form a protective seal with the gingival tissue to resist bacterial invasion and maintain a good environment for osseointegration [3]. Repeated screwing out of the abutment (e.g. implant-level impressions) and fragile soft tissue structures around implants (Fig. 1A) can lead to the breakdown of the biological seal, with bacteria forming the oral cavity reaching the implant–bone interface through the weak points and causing chronic inflammation [1, 4]. According to statistics, the survival rate of dental implant restorations at 10–15 years exceeds 89%, while the incidence of peri-implantitis or peri-implant infection reach 8.9–43.3% and have become an important cause of implant failure [5, 6]. Therefore, good biocompatibility, bioactivity, and antimicrobial properties of abutment materials are the basis for good biological sealing and clinical success of dental implants [1, 7].

Polyetherketoneketone (PEKK) materials have been widely used in the fields of spinal orthopaedics, bone trauma recovery, and joint replacement because of their good thermostability and biocompatibility, mechanical properties similar to those of human bone, radiolucency, and metal-free properties [8, 9]. In addition, their application in dentistry, such as in implants, posts, temporary crowns, and other restorative materials, has been preliminarily verified by research [9–11]. However, PEKK has a biologically inert surface with no specific antimicrobial capability [7, 12], which impedes PEKK materials from forming a close and healthy integration with the surrounding soft tissues. Therefore, its biological properties for use in implant abutment materials need to be improved.

As a high-performance polymeric material, PEKK exhibits strong acid and alkali corrosion resistance and reacts readily with high concentrations of sulphuric acid to form micropores of various sizes on the surface [13, 14]. This surface structure may facilitate the adhesion of osteoblasts; however, whether it facilitates the adhesion of gingival fibroblasts or oral bacteria remains unknown [15, 16]. Some studies have proposed surface coating modification of PEKK materials, which can improve their bioactivity or antimicrobial properties for a short time [17–19]. However, the coating is prone to peeling from the substrate after being subjected to large shear forces or chemical attacks [18, 20]. In addition, studies have also proposed the fabrication of special structures on the surfaces of PEKK materials; however, such methods require sophisticated technology and equipment, and can currently only process pure PEKK instead of PEKK composites [21, 22]. Furthermore, the component blending method proposed by other researchers can easily affect the overall mechanical properties of PEKK, while the modification effect is limited [23, 24]. Therefore, it is necessary to establish a reliable method for modifying PEKK materials.

Plasma immersion ion implantation (PIII) is a non-line-of-sight modification method which uses a high flux current to ionize and vaporize the target material and then implants the ions into the substrate surface through an accelerated electric field (Fig. 1B) [1, 25]. This technique allows the introduction of almost any element (e.g. biotrophic elements) into different types of biomedical substrates [25–27]. The physicochemical properties of the substrate surface can be altered by introducing different chemical elements without peeling [28, 29]. In addition, some of the implanted ions can gradually precipitate from the substrate with the dissipation of energy and produce corresponding biological effects [30, 31]. Magnesium ion (Mg^2+^) is one of the major cations in the human body and has been proven to be an essential element in the regulation of various intracellular processes, such as cell signalling, enzyme activity, energy metabolism, and protein synthesis [30, 32]. Silver (Ag) is a recognized antimicrobial agent; its ions (Ag^+^) and compounds are toxic to some bacteria, viruses, and fungi, but are harmless to humans at safe concentrations [33, 34]. Therefore, the implantation of Mg/Ag dual ions into the surfaces of PEKK materials is expected to combine the biological advantages of both types of ions while improving the bioactivity and antimicrobial properties of the material.

In this study, a glass-fibre-reinforced PEKK composite (PEKK-GF), which shows good aesthetic, biocompatibility, and elastic modulus (16–21 GPa) that can match those of cortical bone (12–20 GPa) better than pure PEKK (4–5 GPa) [9, 14, 35, 36], was selected as the substrate. Furthermore, functional Mg and Ag ions were implanted into the surface of PEKK-GF by PIII, which changed the hydrophilic and electrochemical environment of the sample surface and enabled the release of Mg^2+^ and Ag^+^. The combined effect of these changes promoted the adhesion and proliferation of human gingival fibroblasts seeded on the PEKK-GF surface while inhibiting those of *Streptococcus mutans* and *Porphyromonas gingivalis*. The application of PEKK-GF to dental implants is believed to be broadened and expedited by Mg/Ag PIII modification.

## Materials and methods

### Materials

PEKK-GF samples were provided by Tianfu Industrial Design (China). Mg and Ag cathode sources were provided by Borui Tiancheng Technology (China). Dulbecco’s modified Eagle’s medium (DMEM), foetal bovine serum (FBS), phosphate-buffered saline (PBS), and penicillin/streptomycin were provided by Gibco (USA). The Cell Counting Kit-8 (CCK-8) was provided by Dojindo (Japan). Live/dead viability/cytotoxicity kit, hemin, vitamin K and 4′,6-diamidino-2-phenylindole (DAPI) were provided by Solarbio (China). The MiniBEST Universal Kit, PrimeScript™ RT Kit with gDNA Eraser, and SYBR^®^Premix Ex TaqTM II kit were provided by Takara (Japan). Primary antibodies were purchased from HuaBio (China). Secondary antibodies were provided by Beyotime (China). Ethanol solution was provided by Jinshan Chemical Test (China). Triton X-100, Tween20, paraformaldehyde, glutaraldehyde, phenazine methosulphate, and bovine serum albumin (BSA) were provided by Sigma-Aldrich (USA). Brain heart infusion (BHI) was purchased from OXOID (UK). XTT was purchased from Abcam (UK). The live/dead BacLight bacterial viability kit was provided by Thermo Fisher Scientific (USA). Agar was provided by BIOFROXX (Germany). Sheep blood plates were purchased from Huan Kai Microbial (China).

### Preparation and characterization of the Mg/Ag PIII-modified PEKK-GF

#### PIII treatment

PEKK-GF containing 30% wt of E-glass fibres (aluminium-borosilicate glass, Na_2_O <2%, length 200–350 μm, diameter 25–70 μm) was used in present study. According to previous research of the authors, the compressive strength and modulus of the PEKK-GF samples were 198 Mpa and 20.23 Gpa. Disc-shaped PEKK-GF samples (diameter: 8 mm, thickness: 2 mm) were prepared, and all samples were highly polished before treatment using an ion implantation machine (BPR1, Borui Tiancheng Technology, China) with the parameters shown in Table 1. All samples were grouped based on the type of ion implantation: (i) untreated control group, (ii) Mg PIII group implanted with Mg for 90 min, (iii) Ag PIII group implanted with Ag for 90 min, and (iv) Mg/Ag PIII group implanted with Ag for 90 min and then with Mg for 90 min.

**Table 1.** Parameters of PIII used in this study

Cathode source	Voltage pulse duration (µs)	Pulsing frequency (Hz)	Ion implantation voltage (kV)	Pressure (Pa)	Ion implantation time (min)
99.99% pure Ag	500	5	−20	5×10^−3^	90
99.99% pure Mg	500	5	−15	5×10^−3^	90

#### Surface morphology and roughness of the Mg/Ag PIII-modified PEKK-GF

The morphology of the sample surfaces was first detected using an optical microscope (OM), and the microscopic morphology of the specimen surfaces was then detected using a scanning electron microscopy (SEM, JSM-IT500LA, JEOL, Japan). The roughness of the samples at the microscale level was detected using a laser confocal 3D profilometer (RETC Instrument, USA) with a scanning area of 5 × 5 μm, and calculated using the Gwyddin 2.30 software; the roughness of the samples at the nanometre level was detected using an atomic force microscope (AFM, Shimadzu, Japan) with a scanning area of 500 × 500 nm and calculated using the SPM-9700 software. Five areas in each sample were randomly selected for testing (*N* = 5).

#### Surface wettability and element distribution

A water contact angle measurement machine (SDC-200S, SINDIN, China) was used to determine the wettability of the specimens. A 2-μl drop of distilled water was applied to the clean sample surface with a camera burst interval of 10 ms. The first photo of the liquid forming a stable form on the material was selected, and the contact angle of the droplet was measured using SDC-200S software. Two regions in each sample were randomly selected for testing (*N* = 5).

The distribution of the elements on the sample surface was detected using energy dispersive spectroscopy (EDS, JSM-IT500LA, JEOL, Japan). The detection area was set to 60 × 60 μm and processed using the SEM operation software after scanning. Two areas of the pure matrix and two areas containing glass fibres were randomly selected for testing in each sample (*N* = 3).

#### Detection of Giovanni effect

The chemical valences of the elements on the PEKK-GF surface were determined using an X-ray photoelectron spectroscopy (XPS, AXIS Ultra DLD, Kratos, UK). The detection elements were set as Mg for the Mg PIII group, Ag for the Ag PIII group, and Mg and Ag for the Mg/Ag PIII group. An Al Kα-ray source with 16 kV voltage, 14.9 mA current, 650 μm spot beam diameter, and 100 eV full spectral fluence energy was used. In addition, all the samples were subjected to charge calibration with the standard of C(1s)=284.8 eV. The results were analysed using Thermo Avantage software. Three regions of the pure matrix were randomly selected for testing each sample (*N* = 3).

Dynamic potential polarization curves were obtained using a three-electrode electrochemical workstation (PGSTA T204, Metrohm, Switzerland). The counter electrode was platinum, the reference electrode was silver/silver chloride and the conducting solution was a 5% NaCl solution. The data were analysed using NOVA software (*N* = 3).

The ion release from the samples was detected using an inductively coupled plasma spectrometer (ICP, Agilent-7900, USA). All specimens were cleaned and dried; sterile gauze balls were used to gently wipe the surface of the PIII-treated material to remove some of the oxide films, and the specimens were then put in 10 ml of PBS for the following immersion times: 1, 3, 5, 7, and 14 days. The untreated sample and pure PBS groups were set at the same time. Three times of this test was repeated (*N* = 5).

### *In vitro* biocompatibility of Mg/Ag PIII-modified PEKK-GF

#### Culture and viability

This study was approved by the ethics committee of the university (WCHSIRB-D-2020-448). Primary human gingival fibroblasts (HGFs) were isolated from healthy gingival biopsies of individuals using the outgrowth technique and cultured in DMEM involving 2 mM glutamine, 10% FBS and 100 U/ml penicillin/streptomycin under the condition of 5% CO_2_, 95% humidity and 37°C [1, 3]. Cells from passages 3–6 were used for subsequent tests.

The HGFs viability was investigated using a live/dead viability/cytotoxicity kit. HGFs and PEKK-GF specimens were co-cultured in 48-well plates (density: 2.5 × 10^4^/well) for 24 h. All samples were then incubated in the live/dead solution (Calcein-AM: 2 μM, PI: 4 μM) at 37°C for 30 min protected from light. Finally, the specimens were observed using a confocal laser scanning microscope (CLSM; FV3000, Olympus, Japan), and three areas were randomly selected from each sample for photography (*N* = 5). The ImageJ software was used for cell counting, and the relative content of live and dead cells was calculated as a percentage.

#### Cell proliferation

The CCK-8 assay was conducted to evaluate the proliferation of HGFs. HGFs and PEKK-GF specimens were co-cultured in 48-well plates (density: 2.5 × 10^4^/well) for 1, 3, 5, and 7 days. At each time point, specimens were incubated in the CCK-8 solution at 37°C protected from light (*N* = 5). After 2 h of incubation, the absorbance of the solution in each well was determined at 450 nm using a microplate spectrophotometer (Thermo Scientific Varioskan Flash, USA).

### Adhesive behaviour of human gingival fibroblasts on Mg/Ag PIII-modified PEKK-GF

#### Initial adhesion

The number of adhesive HGFs on the specimens in the initial seeding period was evaluated using DAPI staining. HGFs were seeded on the PEKK-GF samples (density: 2.5 × 10^4^/well) and incubated in 48-well plates for 1, 4, and 24 h, respectively. At each time point, specimens were first immersed in 4% paraformaldehyde at 4°C for 30 min and then incubated in DAPI solution at 37°C for 5 min protected from light. The samples were observed using CLSM and five regions of each specimen were randomly selected for photography (*N* = 5). The obtained images were analysed by the ImageJ software to determine the number of cells adhering to the specimen (Ac), and the area of the photographed region was calculated according to the scale (Aa). The number of cells seeded per well (Tc) and the bottom area of the well plate (Ta) were known. The cell adhesion rate (AR) of the tested samples was calculated based on the following equation:


$$\mathrm{AR}=\frac{\mathrm{Ac}/\mathrm{Aa}}{\mathrm{Tc}/\mathrm{Ta}}$$

#### Scanning electron microscopy

The morphology and distribution of the HGFs were analysed using SEM. HGFs and PEKK-GF specimens were co-cultured in 48-well plates (density: 2.5 × 10^4^/well) for 24 h. The samples were then fixed in 2.5% glutaraldehyde at 4°C for 4 h, after which the specimens were dehydrated by immersing in ethanol solutions with gradient concentrations (30–100%). The specimens were coated with gold before being observed by SEM.

#### Quantitative polymerase chain reaction

The relative expression of some genes in the HGFs was detected using quantitative polymerase chain reaction (qPCR). HGFs and PEKK-GF specimens were co-cultured in 48-well plates (density: 2.5 × 10^4^/well) for 24 h. Total RNA was obtained using the MiniBEST Universal Kit and cDNA was generated using the PrimeScript™ RT Kit with gDNA Eraser according to the manufacturer’s instructions. The genes assessed in this study included focal adhesion kinase (*FAK*), vinculin (*VCL*), fibronectin 1 (*FN1*), integrin β1 (*ITGB1*), integrin α2 (*ITGA2*), collagen type 1 α1 (*COL1A1*), and *GAPDH*. In addition, specific gene primers were synthesized by Tsingke Biotechnology (China); the details are shown in Supplementary Table S1. Finally, qPCR was conducted using the SYBR^®^Premix Ex TaqTM II kit. *GAPDH* was selected as the internal reference gene, and the relative expression levels of the tested genes were measured by the 2^−ΔΔCT^ method (*N* = 5).

#### Immunofluorescence of adhesion-related proteins

HGFs and PEKK-GF specimens were co-cultured in 48-well plates (density: 2.5 × 10^4^/well) for 24 h. Specimens were fixed in 4% paraformaldehyde at 4°C for 30 min and then incubated in the prepared blocking solution at 37°C for 1 h. Following that, specimens were incubated in specific primary antibodies overnight at 4°C and then in the secondary antibodies at 37°C for 1 h protected from light. Finally, the specimens were incubated in the DAPI solution at 37°C for 5 min and observed by CLSM. The samples were washed 2–3 times with PBST between each of the aforementioned two steps. The compositions of the solutions used in present assay are provided in Supplementary Table S2. Three areas per sample were randomly selected for imaging (*N* = 3).

### Antibacterial property of Mg/Ag PIII-modified PEKK-GF

#### Culture and metabolic activity

*Streptococcus mutans* (S.m, UA159) and *P.gingivalis* (P.g, W83) were used for antibacterial tests. S.m was cultured in BHI liquid medium under the air atmosphere of 5% CO_2_ at 37°C, whereas P.g was cultured in BHI liquid medium containing 1 μg/ml vitamin K and 0.0005% hemin under anaerobic atmosphere of 80% N_2_, 10% CO_2_, and 10% H_2_ at 37°C.

Bacterial metabolic activity was evaluated using the XTT reduction assay. S. m. and P. g. were co-cultured with the PEKK-GF samples respectively in 48-well plates (density: 5 × 10^5^ CFU/well) for 24 h. The samples were incubated in BHI liquid medium containing 150 mg/ml XTT and 10 mg/ml phenazine methosulphate for 3 h protected from light. Finally, a microplate spectrophotometer was used to determine solution absorbance of each well at the wavelength of 490 nm (*N* = 5).

#### Fluorescence staining

The live and dead bacteria on the PEKK-GF samples were quantified using the live/dead BacLight bacterial viability kit. Bacteria and PEKK-GF samples were co-cultured in 48-well plates (density: 5 × 10^5^ CFU/well) for 24 h. The samples were incubated in a solution containing 2.5 μM SYTO 9 dye and propidium iodide dye for 15 min protected from light. Three areas of each sample were randomly selected for observation and photography using CLSM (*N* = 3). The ImageJ software was used for counting, and the relative content of live and dead bacteria was calculated as a percentage.

#### The spread plate test

S. m. and P. g. were incubated with the specimens respectively at a density of 1 × 10^6^ CFU/well for 24 h. After being washed twice with PBS, the specimens were placed in 2 ml PBS, and subjected to 5 min of ultrasonic washing and 1 min of vortex mixing to fully detach the bacteria from the materials. Then, the well-mixed bacterial suspensions were serially diluted at 10^−3^, 10^−4^, 10^−5,^ and 10^−6^, and 100 μl of each concentration was plated in triplicates onto the BHI agar plates (S.m) and defibrinated sheep blood plates (P.g). After incubation for 2–3 days, the colony-forming units on the plates were counted to calculate the number of viable bacteria (CFU/ml), and the antibacterial rates (Ra) for different samples were calculated according to the National Standard of China GB 21551.2 protocol [37]. Specifically, the Ra was calculated using the following formula: (Ra) (%)=(A−B)/A × 100%, where A represents CFU of the control group, and B represents CFU of the experimental groups (*N* = 3).

#### Scanning electron microscopy

The morphology and distribution of bacteria in the samples were observed using SEM. Bacteria and PEKK-GF samples were co-cultured in 48-well plates (density: 5 × 10^5^ CFU/well) for 24 h. The samples were fixed in 2.5% glutaraldehyde at 4°C for 4 h, after which the specimens were dehydrated by immersing in ethanol solutions with gradient concentrations (30–100%). The specimens were coated with gold before being observed by SEM.

#### Statistical analysis

All of the biological experiments in present study were conducted in triplicates, and the obtained data were first evaluated for normality and homogeneity of variance. Then the statistical significance of the data was determined using one-way ANOVA followed the *post hoc* Tukey, Bonferroni, or Tamhane tests. In addition, *P *<* *0.05 was considered statistically significant. Data analysis was conducted using SPSS software (version 24.0) and plotting was performed using Origin 21.0 software.

## Results and discussion

### Characterization of Mg/Ag-PIII-modified PEKK-GF

#### Surface morphology and roughness

The surface morphology of materials is an important factor that affects their biological characteristics, and the changes in microscale and nanoscale surface roughness may have different effects on cells or bacteria [1, 38]. As a non-line-of-sight modification method, the change in PIII to substrate morphology is affected by factors such as implantation parameters and substrate properties [1, 32].

**Figure 2.**
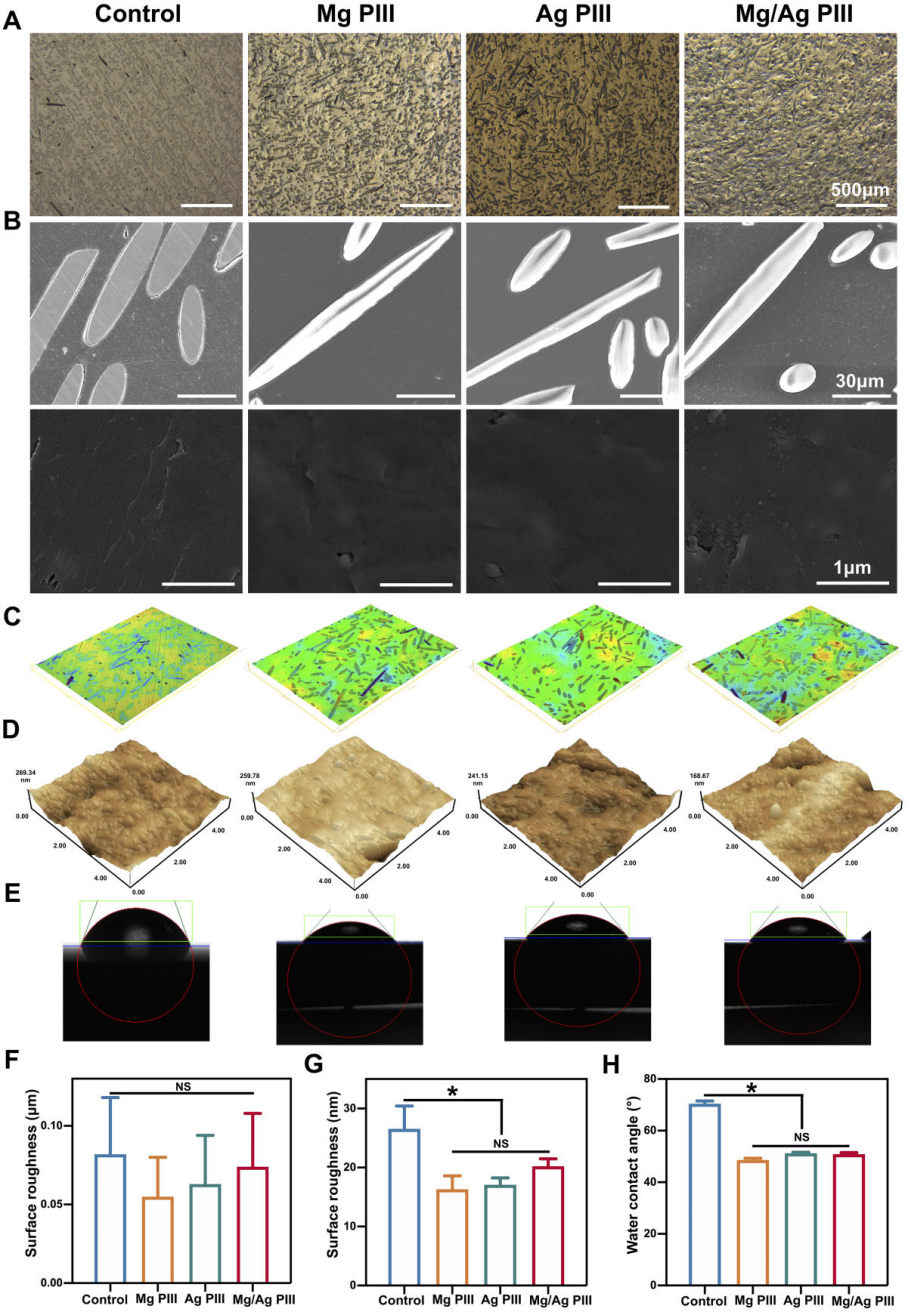
(**A, B**) the OM and SEM micrographs of PEKK-GF samples. (**C, D**) 3D topography and AFM micrographs of the differently treated PEKK-GF. (**E**) Measurements of the wettability. (**F, G**) Surface roughness of the samples detected at the micro and nanoscale. (**H**) Water contact angle measured on the sample surfaces. *P <* *0.05.

Under the optical microscope (Fig. 2A), the surfaces of PIII-treated samples were relatively flat and exhibited a metallic colour, which preliminarily showed the effect of PIII. SEM (Fig. 2B) revealed that the surfaces of the samples before PIII treatment had shallow polishing lines, which were only faintly visible on the surfaces of the PIII-treated samples, and no significant difference was observed in morphology among the PIII-treated groups. The combined SEM results at various magnifications showed that the surfaces of PEKK-GF after PIII treatment were smooth and flat, and there were no obvious morphological changes or new micro/nanostructures.

The 3D profilometer results (Fig. 2C and F) showed that the microscale roughness of the samples after PIII treatment was slightly lower than that before treatment, but the differences were not statistically significant, and no significant difference was observed in roughness among the treatment groups (*P *>* *0.05). AFM results (Fig. 2D and G) showed that the nanoscale roughness of the samples decreased after Mg PIII and Ag PIII treatment (*P *<* *0.05), while no significant changes after Mg/Ag PIII treatment were observed (*P *>* *0.05). According to previous literature, the difference in the roughness of one sample in different size ranges measured by the same or different machines can be several times [38, 39], which is consistent with the present study findings. Generally, the Mg/Ag PIII modification did not cause visibly changes in the macro and micro morphology of the PEKK-GF surface.

#### Surface wettability and element distribution

Detection of wettability (Fig. 2E and H) showed that the water contact angle of all samples was less than 90°. The water contact angle of all specimens decreased after PIII treatment (*P *<* *0.05), while no significant difference was observed in wettability among the treatment groups (*P *>* *0.05). It is known that wettability is influenced by various physicochemical properties of the material surface, and the present results may have been caused by the changes in chemical composition of the PEKK-GF sample surface due to PIII treatment [15, 31].

**Figure 3.**
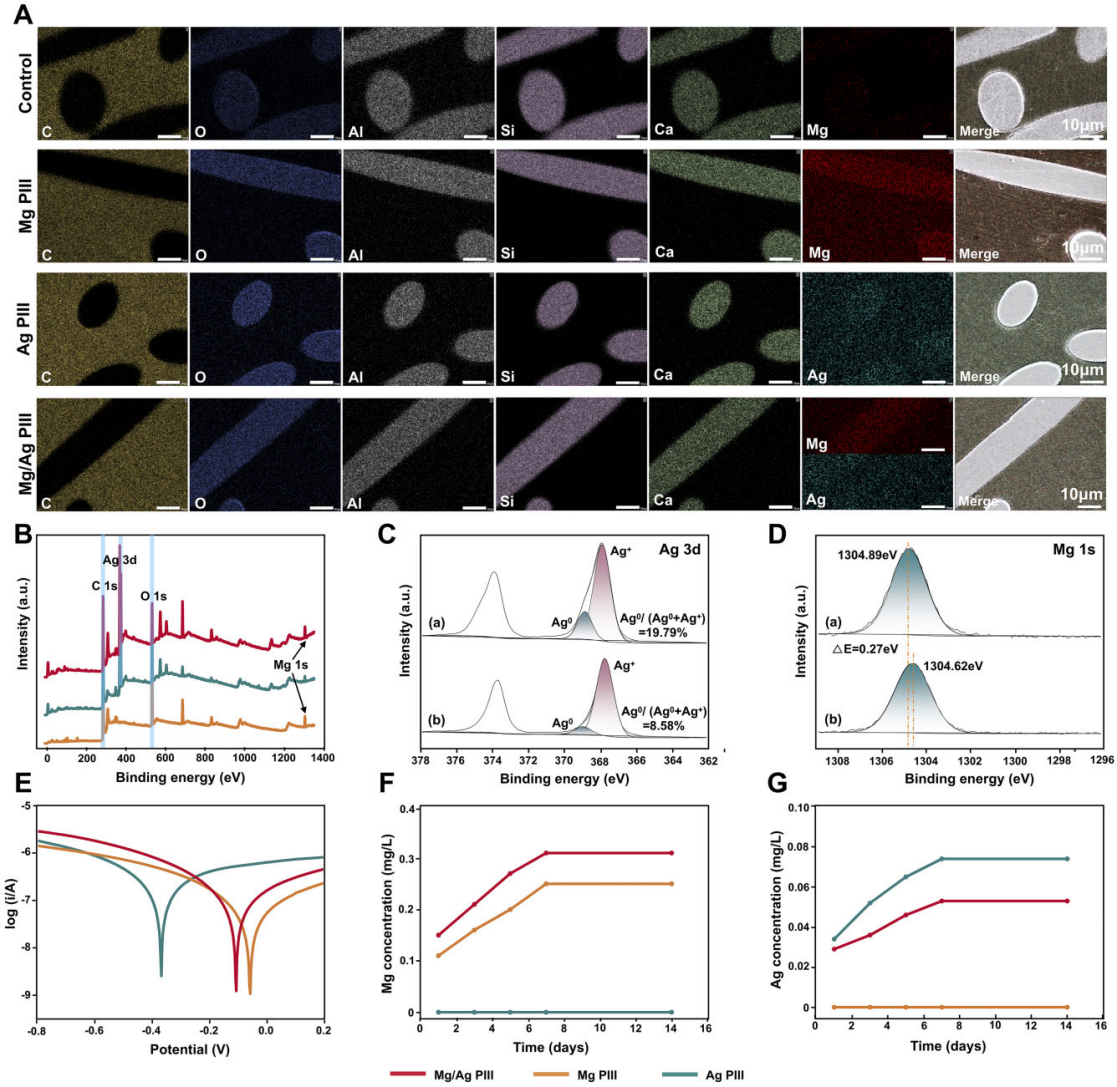
(**A**) Element distribution on the sample surfaces (fibre parts) detected by EDS. (**B**) XPS analysis of the PIII-treated PEKK-GF samples. (**C**) Valence analysis of Ag in different samples, (a): Mg/Ag PIII, (b): Ag PIII. (**D**) Valence analysis of Mg in different samples, (a): Mg/Ag PIII, (b): Mg PIII. (**E**) Potentiodynamic polarization curves of differently treated PEKK-GF. (**F, G**) Mg^2+^ and Ag^+^ released from the samples with original sheet form (diameter: 8 mm, height: 2 mm) immersed in 10 ml PBS for different periods.

The EDS results showed that the elemental composition of the PEKK matrix surface was mainly C and O, suggesting a pure PEKK polymer material (Supplementary Fig. S1). In addition, after PIII treatment, uniform distributions of Mg, Ag, and Mg/Ag were detected on the surface of the specimens, suggesting the successful implantation of the ions into the PEKK surface. Meanwhile, Al, Ca, Si, Mg, C, and O were detected on the surface of the fibre parts before PIII treatment (Fig. 3A). In addition, no Na was detected in the glass fibres, further verifying that the glass fibres used were alkali-free (NaO_2_ content <2%). The distribution of Mg was greater on the surface of the Mg PIII-treated glass fibres than on that of the matrix, which may be related to the Mg content in the glass fibre itself. The distribution of Ag was slightly greater on the surface of the Ag PIII-treated glass fibre than on that of the matrix, which may be related to the difference in the ion implantation caused by the different physicochemical properties of the two materials [1, 25].

#### Giovanni effect

As two different elements were implanted into the PEKK-GF surface, the electrochemical environment on the material surface that cells and bacteria have contact with might have changed. Therefore, further tests were conducted to confirm this change.

Figure 3B shows the results of XPS analysis of the PIII-treated PEKK-GF, further indicating that the successful implantation of Mg and Ag into the PEEK-GF surface. In addition, Fig. 3C shows the XPS peaks of Ag(3d) in the Mg/Ag PIII and Ag PIII groups, where the peaks at ∼368.9 eV and ∼367.8 eV can be assigned to Ag^0^ and Ag^+^, respectively [40, 41]. It can be observed that the ratio of Ag^0^/(Ag^0^+Ag^+^) increased from 8.58% to 19.79% in the Mg/Ag PIII group compared with the Ag PIII group. Figure 3D shows the XPS peaks of Mg(1s) in the Mg/Ag PIII group and the Mg PIII group. The binding energy of Mg(1s) in the Mg/Ag PIII group (1304.89 eV) shifts to a higher electric field than that in the Mg PIII group (1304.62 eV, △E = 0.27 eV). Therefore, it can be speculated that electrons were transferred from Mg to Ag in the Mg/Ag PIII group, which weakened the shielding effect of the outermost electrons of Mg and increased its binding energy. These electrons moved to a higher electric field and were obtained by Ag^+^, increasing the proportion of Ag^0^ [42].

Figure 3E shows the dynamic potential polarization curves of the PIII-treated specimens. The corrosion potential of the Mg/Ag PIII group shifted to the negative side compared with that of the Mg PIII group, suggesting that the latter group had better corrosion resistance. In addition, ICP results (Fig. 3F and G**)** indicated that all the PIII groups showed ion release, and the release peak occurred on the 5th to 7th days. The maximum release of Mg^2+^ in the Mg PIII group (0.25 mg/l) was lower than that in the Mg/Ag PIII group (0.31 mg/l), while the maximum release of Ag^+^ in the Mg/Ag PIII group (0.053 mg/l) was slightly lower than that in the Ag PIII group (0.074 mg/l).

Based on the XPS, dynamic potential, and ICP results, it can be concluded that there was an electrochemical corrosion effect—the Giovanni effect—between the two implanted elements in the Mg/Ag PIII group. Specifically, when there is a potential difference between two types of metals, an electric current can be generated through the medium, followed by an electrochemical reaction that causes the anode to lose electrons and be oxidized [43, 44]. The Giovanni effect has been applied in the modification of biomaterials as the electrochemical effect can help modulate different biological effects on cells and bacteria and shows great potential in the field of biomaterials [30, 45]. In the present study, the Giovanni effect was considered to play an important role in increasing the release of Mg^2+^ while reducing the release of Ag^+^.

### *In vitro* biocompatibility

The soft tissue attachment around implant abutments is mainly composed of HGFs and collagen fibres, which account for about 1/3 and 2/3 of the volume, respectively. In addition, collagen fibres are mainly secreted by HGFs for gingival wound healing and tissue regeneration [1, 7]. Therefore, the abundance and viability of HGFs on abutment surfaces are essential to the formation of healthy peri-implant biological seals.

**Figure 4.**
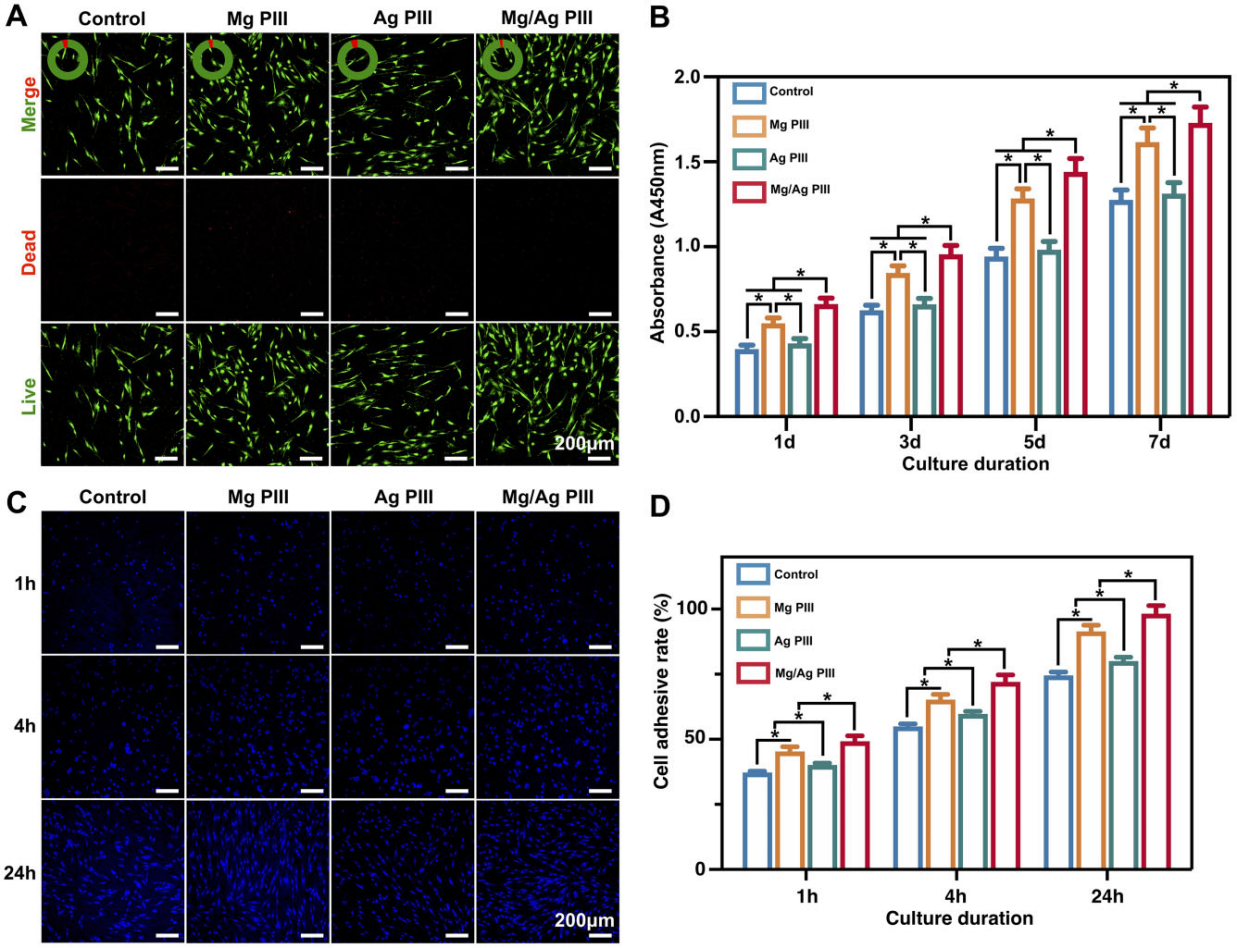
(**A**) Live/dead fluorescent images of HGFs cultured on different PEKK-GF samples, indicating live (green) and dead (red) cells. (**B**) Results of the CCK-8 assay evaluating the proliferation activity of HGFs co-cultured with the samples for 1, 3, 5 and 7 days. (**C, D**) Initial adhesion of HGFs on the PEKK-GF samples after seeding for 1, 4 and 24 h. (C) CLSM images of cell nuclei stained with DAPI and (D) their quantitative results. For each time point, *P <* *0.05.

Results of live/dead cell fluorescence staining (Fig. 4A) showed that all groups had a small percentage of dead cells. Although there seemed to be some differences in the density of adherent HGFs on the PEKK-GF samples from different groups, no significant difference was observed in the relative content of dead cells among these groups (*P *>* *0.05), suggesting that all groups showed good biocompatibility.

The CCK-8 assay (Fig. 4B) showed that cell number of all groups increased with increasing incubation time. Cell proliferation was the highest at all time points in the Mg/Ag PIII group, followed by the Mg PIII group, suggesting that Mg implantation promotes the proliferation of HGFs. In contrast, the proliferation of HGFs in the Ag PIII group and the control group was comparable at all time points (*P *>* *0.05).

### Adhesive behaviour of HGFs

Timely and sufficient adhesion of cells on the sample surface is important for the formation of good integration of soft tissue with the material. The early adhesion of HGFs to the specimens was detected using DAPI staining. As shown in Fig. 4C and D, the cell adhesion rate increased with increasing incubation time. At each time point, the cell adhesion rate of the Mg PIII and Mg/Ag PIII groups was significantly higher than that of the other two groups, and the increase became more prominent with incubation time. In addition, the cell adhesion rate was slightly higher in the Ag PIII group than in the control group (*P *<* *0.05).

**Figure 5.**
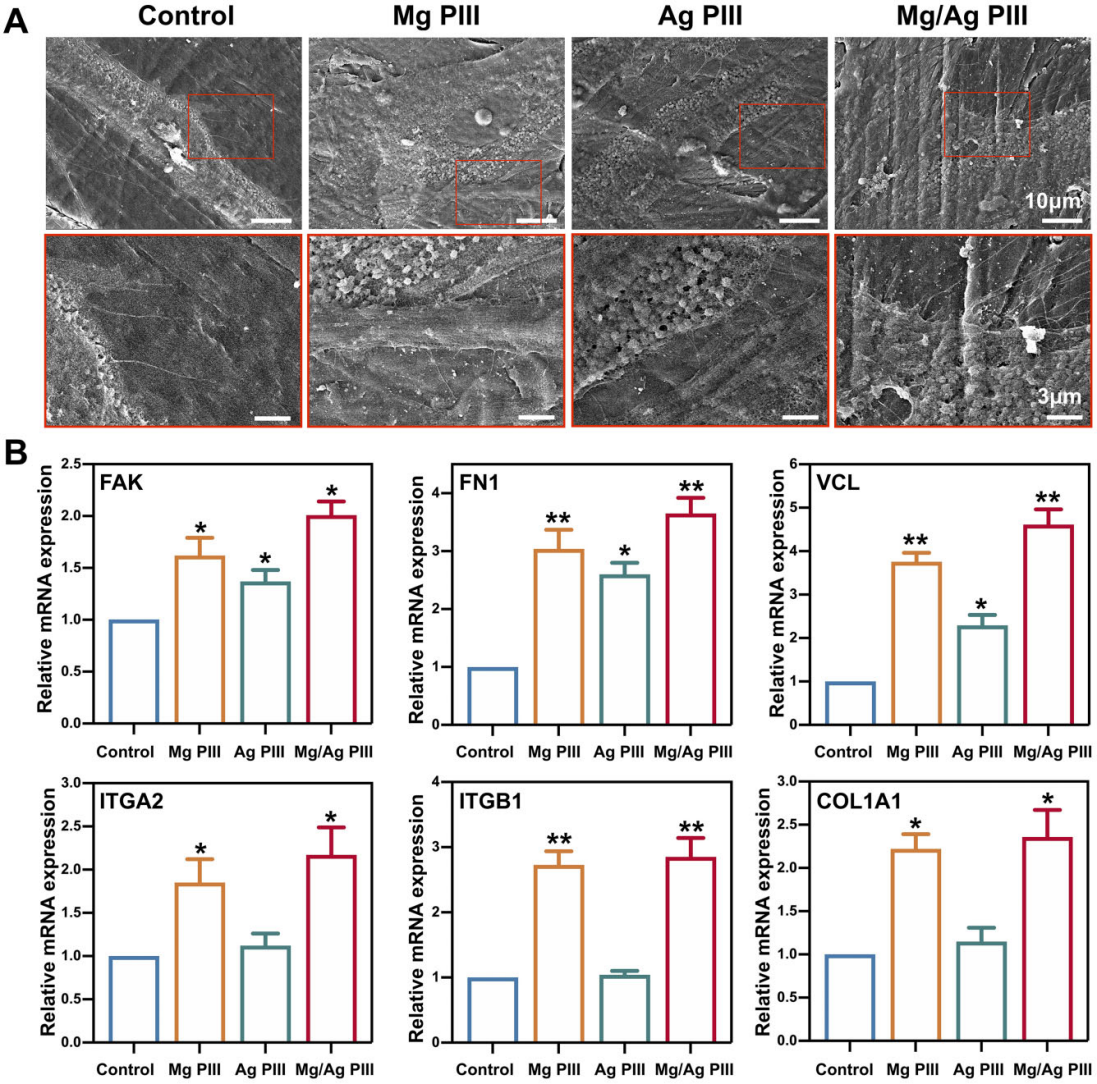
(**A**) SEM micrographs showing the morphology of cells adhered to the PEKK-GF samples after incubation for 24 h. (**B**) qPCR results showing the expression of adhesion-related genes in HGFs cultured on PEKK-GF for 24 h, *P <* *0.05, **P <* *0.01 when compared with the control group.

SEM images are shown in Fig. 5A. After 24 h of incubation, the HGFs in all the groups were uniformly distributed and well-extended on the specimens. In particular, HGFs in the control group were mainly spindle-shaped or elongated with a number of filopodia around the cell body. HGFs in the Ag PIII group were mostly elongated, with more protrusions and filopodia than those in the control group. In contrast, HGFs in the Mg/Ag PIII and Mg PIII groups were mainly elongated or polygonal and became more dispersed and flattened on the specimens than in the other two groups. They also had widely extended cytoplasmic protrusions and a large number of filopodia, which were tightly bound to the substrate. In addition, good intercellular junctions were observed in the cells of the Mg/Ag PIII group.

To figure out the molecular mechanism of cell adhesion behaviour on the sample surface, the expression of adhesion-related genes in HGFs, including *FAK*, *VCL*, *ITGB1*, *ITGA2*, *FN1* and *COL1A1*, was detected by qPCR [1]. As shown in Fig. 5B, after 24 h of incubation, the expression of these genes was significantly higher in HGFs of the Mg/Ag PIII and Mg PIII groups than in those of the other two groups. In addition, HGFs in the Ag PIII group showed higher mRNA expression of *FAK*, *FN1*, and *VCL* compared to the control group, which was in line with the results of the early adhesion test, wherein cell adhesion rate was slightly higher in the Ag PIII group than in the control group.

**Figure 6.**
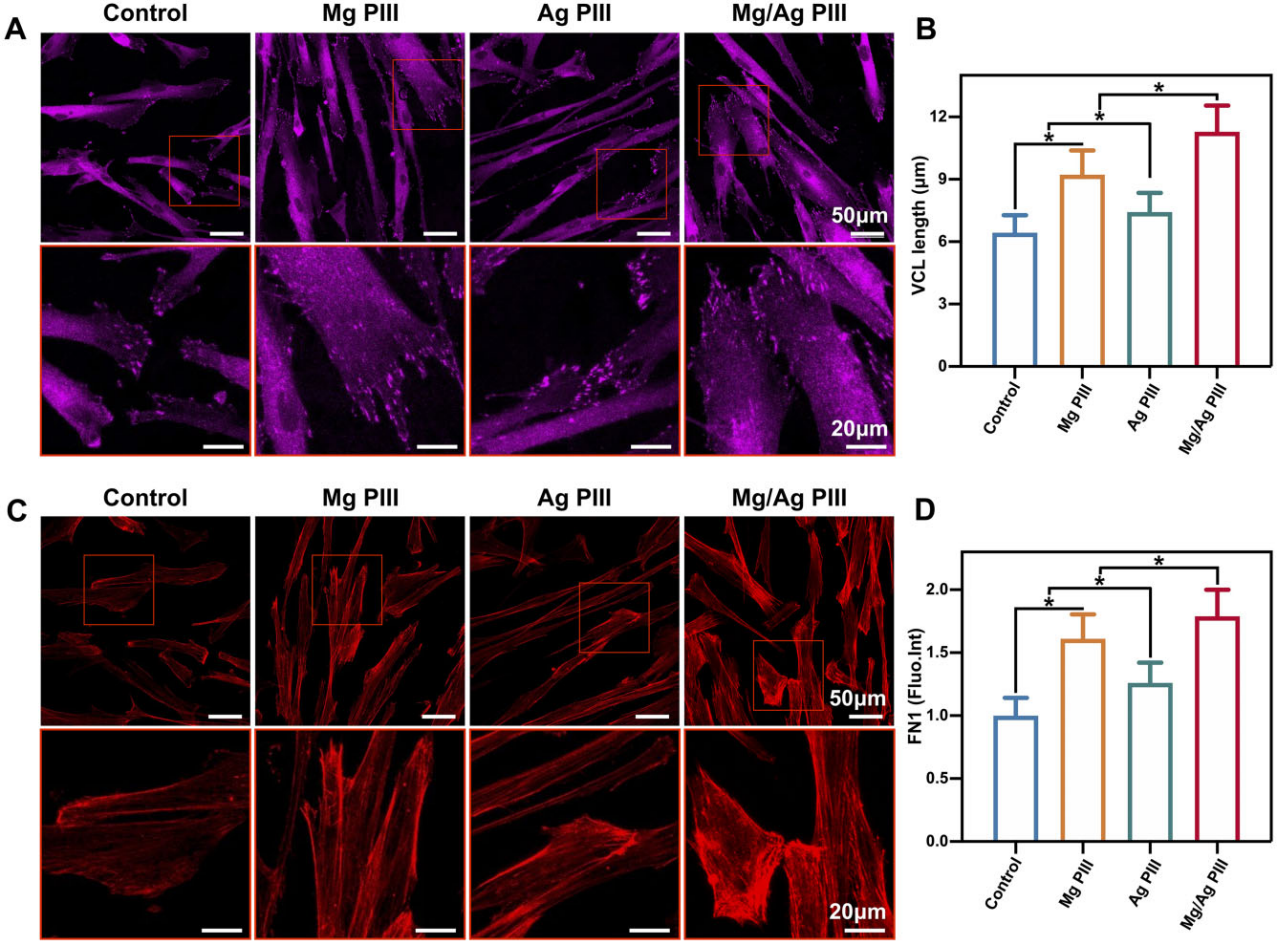
Immunofluorescent images showing the expressions of vinculin which localizes at focal adhesion sites (**A**), and the expressions of fibronectin 1 (**C**) in HGFs after incubation for 24 h. (**B**) The length of vinculin points of HGFs. (**D**) The relative fluorescent intensity of fibronectin 1 in HGFs. *P <* *0.05.

In addition, protein expression of VCL and FN1 was detected using immunofluorescence staining. VCL is mainly located at the adhesion site and is associated with cell adhesion strength and migration [32]. As shown in Fig. 6A and B, HGFs in the Mg/Ag PIII and Mg PIII groups expressed more VCL than the other two groups, while cells also showed slightly higher VCL expression in the Ag PIII group than in the control group. FN1 is one of the most important matrix adhesion proteins that can connect collagen networks to cytoskeletal microfilaments via transmembrane receptors such as integrins [30, 32]. As shown in Fig. 6C and D, HGFs in the Mg/Ag PIII and Mg PIII groups expressed more extensively expanded FN1 than those in the other two groups. Moreover, the expression of FN1 in cells of the Ag PIII group was higher than that those of the control group. The observed differences in immunofluorescence among the PIII-treated groups were consistent with the qPCR results.

### Mechanism underlying the effects of the Mg/Ag PIII-modified PEKK-GF on HGFs

Material characterization established that PIII treatment caused no significant morphological changes in PEKK-GF. Therefore, the better proliferation and adhesion of HGFs on the samples of the Mg/Ag PIII and Mg PIII groups may be partly attributed to the presence of Mg. Okawachi *et al.* [46] showed that titanium hydrothermally treated with an Mg solution showed better adhesion integration with Sa3 epithelial cells and NIH3T3 fibroblasts. Amberg *et al.* [47] found that Mg^2+^ increases vinculin expression in fibroblasts. In a study by Zhu *et al.* [32], the expression of integrin α5, β1, and β3 subunits was found to be significantly increased in HGFs co-cultured with Mg PIII-treated titanium samples, and integrins further mediated cell motility and adhesion by activating widely distributed adhesion molecules, promoting the adhesion, proliferation, and migration of HGFs on Mg PIII-treated titanium surfaces. Our findings are in line with those of the aforementioned studies. The maximum concentration of Mg^2+^ in this study (0.32 mg/l) did not reach close to that in Zhu’s study (0.98 mg/l), which may be related to the differences in substrate properties (PEKK-GF vs Ti), as well as the conditions and parameters of ion implantation [30, 31]. The promotion of adhesion and proliferation of HGFs was similar in the two studies likely because the rate of Mg^2+^ release was faster in this study, peaking at Days 5–7 (0.32 mg/l), while the release of Mg^2+^ was slower in Zhu’s study, with the concentration at Day 7 was 0.38 mg/l [32]. Moreover, the *in vitro* tests were conducted over a short period (within 7 days) and may have played a role in similar conclusions from the two studies.

In addition to producing the release of Mg^2+^, Mg PIII treatment also improved the wettability of PEKK-GF, which was demonstrated to upregulate the expression of cell adhesion molecules and enhance intercellular signalling [48]. Gu *et al*. [49] and Li *et al.* [50] found that the PI3k/AKT signalling pathway, which is a crucial downstream component of FAK and integrins, can be significantly influenced by material wettability and mediates the signal transduction mechanism that controls cell proliferation, migration, and extracellular matrix secretory activities. Therefore, improved surface wettability may be another reason for the promotion of the adhesion and proliferation of HGFs on PEKK-GF.

HGFs also exhibited better biological behaviours in the Mg/Ag PIII group than in the Mg PIII group, which may be attributed to the Giovanni effect existed between Mg and Ag [45]. The standard electrode potential of Ag (0.7996 V) is totally different from that of Mg (−2.363 V). Thus, the corrosion or the ion release process in the Mg/Ag PIII group can be controlled by the Giovanni effect, where Mg acts as an anode and the anodic reaction promotes the release of Mg^2+^ [43, 44]. This is consistent with the results of the ICP test that verified a higher concentration of Mg^2+^ in the Mg/Ag PIII group, which may promote the adhesion and proliferation of HGFs [45]. Ag acts as a cathode, and the cathodic response not only limits the release of Ag^+^ but also consumes the surrounding protons. The altered proton environment is speculated to possibly stimulate the overall energy-dependent response and proliferation of HGFs [43]. which is further discussed in the bacterial experiments section.

The Ag PIII group showed better binding to HGFs than the control group and exhibited good biocompatibility. On the one hand, the minimum safe concentration of Ag^+^ is reported to be 2 μg/ml, which is approximately 27 times the peak Ag^+^ concentration in the present study, suggesting that the Ag PIII group did not exhibit significant biotoxicity [51]. On the other hand, the wettability of PEKK-GF increased significantly after Ag PIII treatment (*P *<* *0.05), which may be responsible for the increased adhesion of HGFs in the Ag PIII group [48]. Of course, the biological effect through increased wettability alone is limited, which explains the Ag PIII group showed lower positive effect on HGFs adhesion than the Mg PIII and Mg/Ag PIII groups [52].

The above mechanism was deduced by considering the results of the material characterization, *in vitro* tests, and previous literature, and understanding the precise molecular mechanism warrants further investigation. The Mg/Ag PIII treatment enhanced the integration of PEKK-GF with HGFs and may provide a reference for the modification of PEKK abutment materials in the future.

### Antibacterial property

#### Antibacterial effect

The oral cavity is a complex environment comprising multiple microbial pathogens. The antimicrobial properties of dental implant restoration material are crucial for the clinical success of dental implant restorations. S.m and P.g are two representative periodontal pathogens which play an important role in peri-implantitis [1, 6]. Therefore, they were selected for tests in the present study.

**Figure 7.**
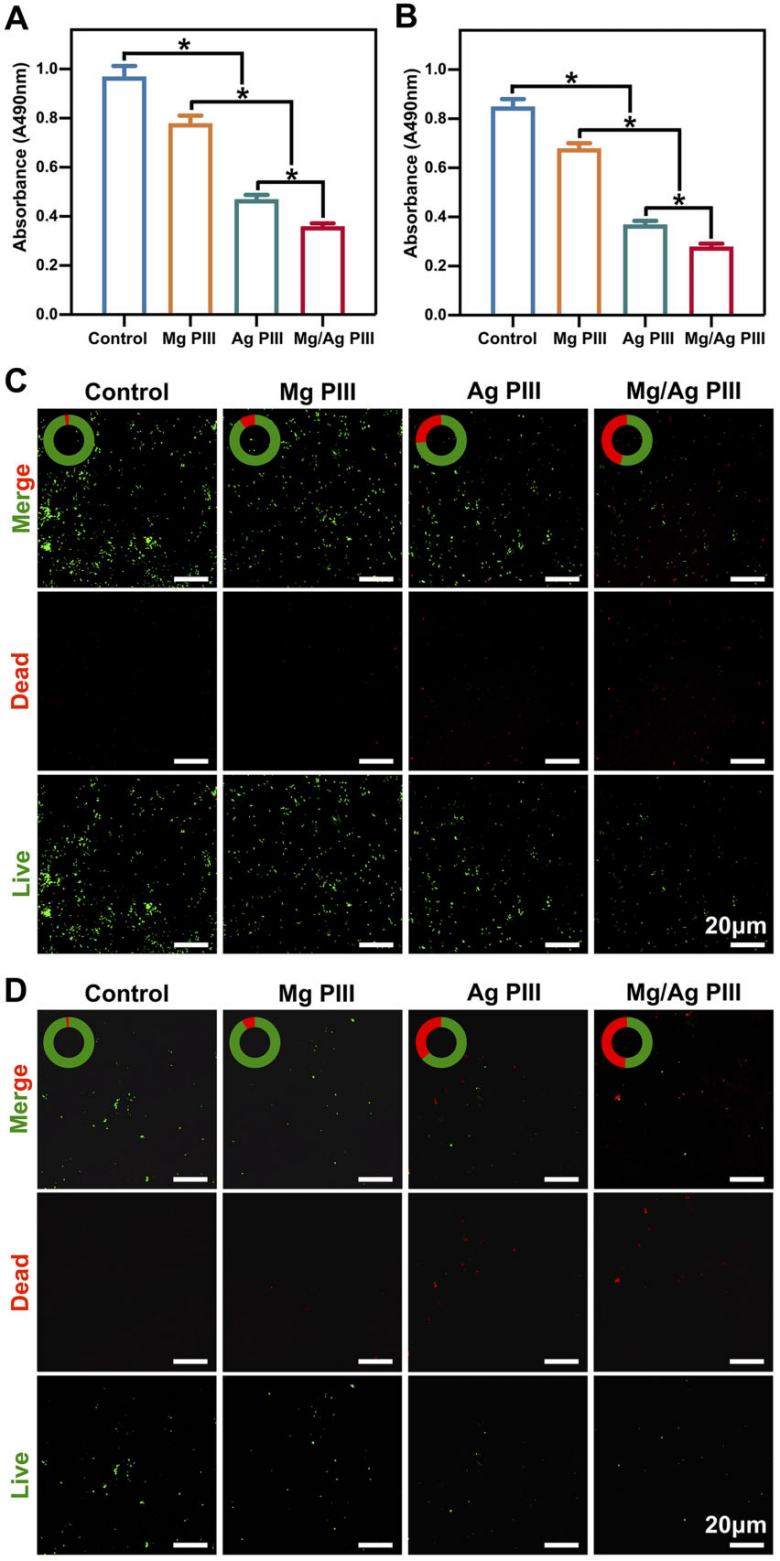
(**A, B**) Metabolic activity of *Streptococcus mutans* (S.m) and *Porphyromonas gingivalis* (P.g) detected using the XTT reduction assay after incubation for 24 h, *P <* *0.05. (**C, D**) Live/dead fluorescent images of S.m and P.g cultured on different PEKK-GF samples for 24 h, indicating live (green) and dead (red) bacteria.

The results of the XTT reduction assay (Fig. 7A and B) revealed that regardless of the type of bacteria, the absorbance of the Mg/Ag PIII and Ag PIII groups was significantly lower than that of the other two groups, suggesting that Ag implantation significantly reduced the metabolic activity of the tested bacteria. In addition, for both S. m and P. g, metabolic viability was slightly lower in the Mg PIII group than in the control group, suggesting that Mg implantation can also help inhibit bacterial metabolism.

The results of live/dead bacteria staining (Fig. 7C and D) showed that after co-culture with the specimens for 24 h, the percentages of dead bacteria were significantly higher in both the Mg/Ag PIII and Ag PIII groups than in the other two groups, suggesting that Ag implantation groups had better antibacterial ability. In addition, for both S.m and P.g, the percentages of dead bacteria were lower in the control group than in the Mg PIII group. Therefore, the antibacterial abilities of the specimens in the PIII-treated groups were verified.

**Figure 8.**
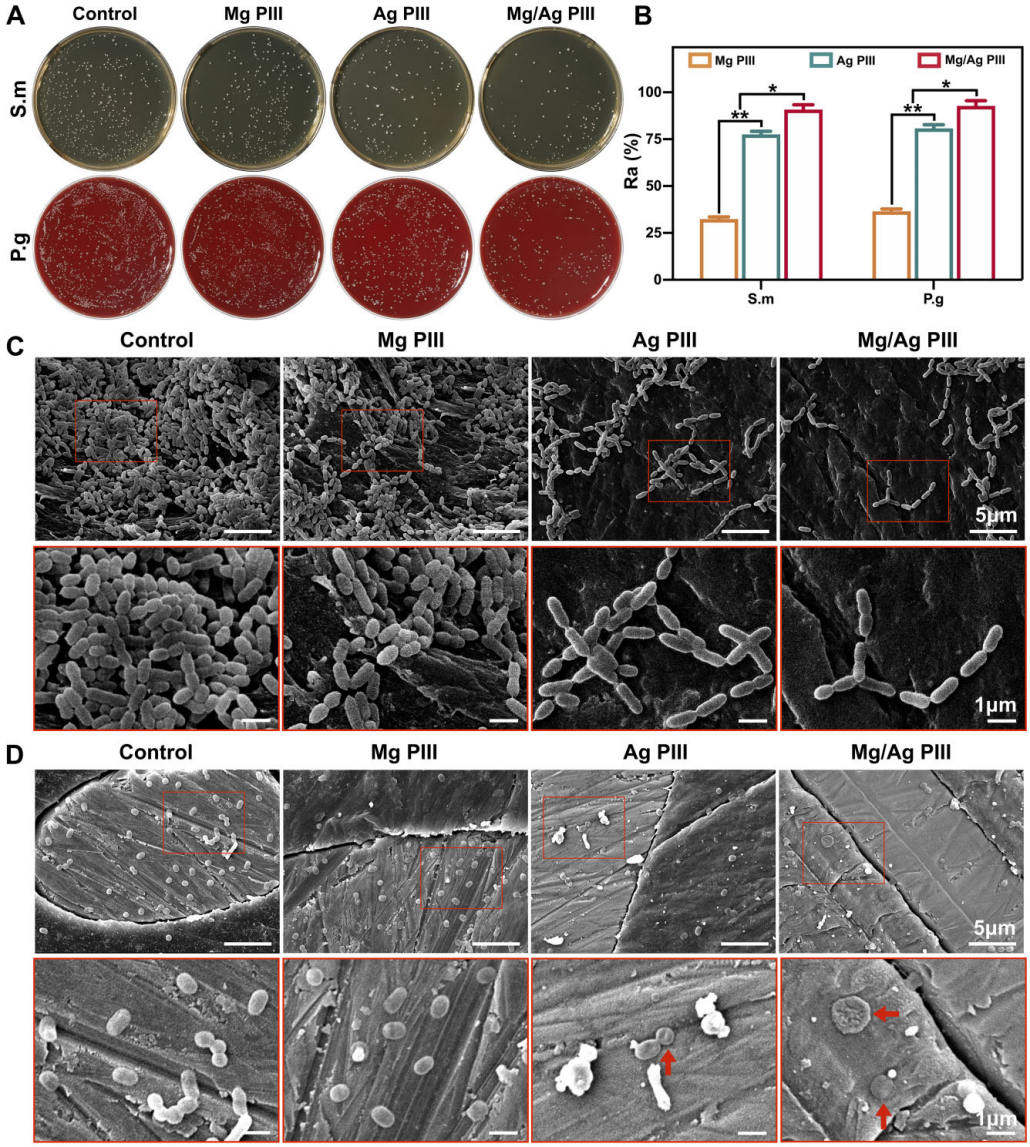
(**A**) Images of re-cultivated S.m and P.g colonies from the PEKK-GF samples after incubation for 24 h. (**B**) Antibacterial rates (Ra) of the samples, *P <* *0.05; **P <* *0.01. (**C, D**) SEM micrographs showing the morphology and distribution of S.m and P.g cultured on the PEKK-GF samples for 24 h (the red arrows indicate the dead bacteria with distorted morphologies). for each bacterium, significant difference was found between groups with dissimilar symbols (*P *<* *0.05).

Figure 8A and B show the results of bacterial colony counting and the Ra values of S.m and P.g on different samples. Both types of bacteria showed significantly lower colonies in the Mg/Ag PIII and Ag PIII groups than in the other two groups. For S.m, the Mg/Ag PIII group exhibited higher Ra value than the Ag PIII group (*P *<* *0.05), and they both showed obviously higher Ra than the Mg PIII group (*P *<* *0.01). For P.g, the Mg/Ag PIII group showed the highest Ra value, while the Mg PIII group showed the lowest Ra. This trend was consistent with bacterial metabolism and live/dead bacterial staining results.

The morphology and distribution of the attached bacteria detected using SEM are shown in Fig. 8C and D. There were more bacteria attached on the surfaces of PIII-treated samples than on those of the untreated samples. S.m aggregated and stacked into grape-like colonies on the surfaces of specimens in the control and Mg PIII groups, and the density of colonies on the Mg PIII samples was slightly lower than that on the untreated samples. The stacking of colonies in the Mg/Ag PIII and Ag PIII groups was significantly reduced, with the long-chain structure was destroyed, and the morphology of some bacteria was changed. P.g were scattered on the specimen surfaces with denser distributions in the control and Mg PIII groups than in the other two groups, and showed rounded or short-rod-like morphology. In the Mg/Ag PIII and Ag PIII groups, P.g were sparsely distributed, and the morphology of some attached bacteria was distorted, shrinking into a round or pie shape.

#### Mechanism underlying the antibacterial property of the Mg/Ag-PIII-modified PEKK-GF

Antibacterial tests revealed that the Mg/Ag PIII and Ag PIII samples had significantly better antibacterial capacity than the other two groups of samples, indicating that Ag may act as a dominant role in the improved antibacterial performance of PEKK-GF. Although Ag is a recognized antibacterial agent, there are various conclusions regarding its specific antibacterial mechanism [53, 54]. Some researchers suggest that the antibacterial abilities of Ag depend on its shape and size. For example, compared with bulk metallic Ag, Ag nanoparticles are more reactive because they own larger active surface area [55]. In addition, Morones *et al.* [56] suggested that only Ag particles with diameters of 1–10 nm directly interact with bacteria [57]. While Chen *et al.* [58] suggested that the antibacterial effect of Ag is determined by the existence of Ag^+^. Some other researches have suggested that oxidizing substances, in place of reducing agents, are the main reason for the antibacterial effect of Ag [59, 60]. In contrast, Su *et al.* [61] found that nanohybrids of Ag and clay could lead to bacterial death only by physical contact, similar to Cao *et al.* [43]. In a study by Zhao *et al.*, the release of Ag^+^, after Ag implantation into a titanium surface, was less than 0.05 mg/l at 28 days, suggesting that the increased antibacterial efficacy should not be attributed to the release of Ag^+^ but the difference in the standard electrode potential between Ag and titanium that causing bacterial death because of the altered electrochemical environment of the material surface they were in contact with Ref. [45]. Specifically, the standard electrode potentials of Ag (0.7996 V) and Ti (−1.630 V) are totally different, and an electrochemical corrosion reaction may occur between the two elements. In this case, Ag acts as the cathode for the reaction, consuming surrounding protons and disrupting ATP synthesis driven by the transmembrane proton electrochemical gradient in the bacterial cytosol [43, 45]. This mechanistic explanation is consistent with that suggested by Qin *et al.* [51].

Based on the above literature and the experimental results in present study, the antibacterial effect of the Ag PIII group may be attributed to the comprehensive effect of various antibacterial mechanisms of Ag, including the release of Ag^+^ and physical contact of bacteria with Ag, which can be partly verified by the results of live/dead bacterial staining and SEM observation. The antibacterial activity of the Mg/Ag PIII samples was improved compared to that of the Ag PIII samples. According to the ICP test results, the amount of Ag^+^ released from the Mg/Ag PIII group was slightly less than that from the Ag PIII group, indicating that the improved antibacterial activity of the Mg/Ag PIII group cannot be attributed to the presence of Ag^+^ alone. Material characterization revealed a Giovanni effect in the Mg/Ag PIII group. In this case, a proton-consuming region is formed near the Ag cathode, which was reported can disrupt ATP synthesis in bacteria [43, 45]. During bacterial respiration, protons are transferred from the cytosol to outside the bacteria through the electron transport chain, thus establishing an electrochemical gradient of protons. ATP synthesis occurs as protons migrate along the electrochemical gradient and enter bacteria via ATPase. Actually, the electrochemical gradient of protons supplies the driving force for ATP synthesis in most bacteria and the mitochondria in eukaryotic cells and is crucial for maintaining energy-dependent reactions [62]. In contrast, proton depletion near Ag disrupts the transmembrane proton electrochemical gradient in bacteria and thus may lead to inactivation of ATP synthesis, ion transport, metabolite chelation, and finally bacterial death [43, 63, 64].

The present study showed that proliferation of HGFs in the Mg/Ag PIII group was enhanced, which was related to not only the increased release of Mg^2+^ but also possibly the formation of proton-depleting regions near Ag caused by the Giavanni effect. Since the volume of eukaryotic cells is much larger than that of prokaryotic cells, and the location of ATP synthesis in eukaryotic cells is the mitochondria with a complex inner membrane structure, ATP synthesis is not easily disturbed by the local proton depletion zone [65, 66]. In contrast, the overall energy-dependent response and proliferation of eukaryotic cells can be facilitated because of the larger proton-accumulating region outside the proton-depleting region [43]. Nevertheless, the precise mechanisms underlying this phenomenon should be further investigated.

The antibacterial activity of the Mg PIII samples was slightly higher than that of the untreated samples. Actually, the antibacterial properties of Mg have been reported previously. Rahim *et al.* [67] showed that bacterial proliferation was inhibited in the presence of metallic Mg and aqueous Mg corrosion extracts, and suggested that the antibacterial effect was related to the increased pH around Mg, creating a local alkaline environment. A detailed analysis of this mechanism showed that, in a biological environment where metallic Mg is exposed to water, Mg can react with H_2_O to produce H_2_, Mg^2+^, and OH^−^ [68]. H_2_ is poorly soluble in biological fluids and can easily diffuse away, whereas soluble Mg^2+^ and OH^−^ can immediately diffuse into surrounding tissues, creating a local alkaline environment [69]. An alkaline environment is reported can consume protons and affect bacterial ATP synthesis, and thus, is not conducive to bacterial survival [67]. However, low-solubility MgOH_2_ precipitates when the localized ion concentration exceeds the saturation limit, which is a protective corrosive layer on the Mg surface in biological fluids and can slow down subsequent corrosion and increase of local pH [68, 69]. In turn, the dissolution of the corrosive layer can lead to an increase in the local Mg^2+^ concentration and pH [70]. Zaatreh *et al.* [71] demonstrated that titanium modified by a rapidly corroding Mg coating could achieve both good biocompatibility and antibacterial properties. However, the antibacterial effect of Mg is dependent on its reaction with the external environment and is influenced by several factors that are difficult to control. Therefore, the antibacterial effect of Mg is inferior to that of Ag, which is recognized as a broad-spectrum antibacterial agent [67, 71]. This mechanism partly explains why the antibacterial activity of the Mg PIII samples was less effective than that of the Ag PIII and Mg/Ag PIII samples.

## Conclusion

In this research, to improve the biological properties of PEKK materials used as implant abutments, dual Mg and Ag ions were energetically introduced into PEKK-GF. The surface characterization of PIII-modified PEKK-GF and its biological effects on HGFs and periodontal pathogens were investigated for the first time. PIII treatment had no obvious impact on the surface morphology of PEKK-GF. The surface wettability and electrochemical environment of PEKK-GF changed after co-implantation and enabled the release of Mg^2+^ and Ag^+^ modulated by Giavanni effect. *In vitro* experiments showed that Mg/Ag PIII-treated PEKK-GF is effective in promoting the proliferation and adhesion of HGFs and upregulating the expression of proteins and genes related to adhesion. Furthermore, the Mg/Ag PIII-treated samples inhibited the metabolic viability and adhesion of *S.mutans* and *P.gingivalis* on their surfaces, distorting bacterial morphology. Therefore, Mg/Ag PIII modification improved the applicability of PEKK-GF as an implant restoration material. Of course, further investigations, such as proteomics experiments and *in vivo* studies, are needed to reveal deeper mechanisms of the biologic effects of Mg/Ag PIII-treated PEKK-GF and expedite its application in dentistry.

## Supplementary Material

rbad066_Supplementary_Data
