# Supplementary material for: Enhancing cell adhesive and antibacterial activities of glass-fibre-reinforced polyetherketoneketone through Mg and Ag PIII
Source: Regen Biomater. 2023 Jul 12;10:rbad066. doi: 10.1093/rb/rbad066 (PMC10363026; doi:10.1093/rb/rbad066)
Supplement: rbad066_Supplementary_Data [file rbad066_supplementary_data.zip › Suppl. Tables.docx]

**Table S1 Details of the gene primer pairs**

| Gene | Primers (5’-3’)  (F = forward; R = reverse) |
| --- | --- |
| FAK | F：GCTTACCTTGACCCCAACTTG  R：ACGTTCCATACCAGTACCCAG |
| VCL | F：CTCGTCCGGGTTGGAAAAGAG  R：AGTAAGGGTCTGACTGAAGCAT |
| ITGB1 | F：CCTACTTCTGCACGATGTGATG  R：CCTTTGCTACGGTTGGTTACATT |
| ITGA2 | F：CCTACAATGTTGGTCTCCCAGA  R：AGTAACCAGTTGCCTTTTGGATT |
| FN1 | F：CGGTGGCTGTCAGTCAAAG  R：AAACCTCGGCTTCCTCCATAA |
| COL1A1 | F：GAGGGCCAAGACGAAGACATC  R：CAGATCACGTCATCGCACAAC |
| GAPDH | F：GGAGCGAGATCCCTCCAAAAT  R：GGCTGTTGTCATACTTCTCATGG |

**Table S2 Components of various solutions**

| Name | Composition |
| --- | --- |
| Blocking solution | PBS containing 5% BSA and 0.3% TritonX-100 |
| Antibody diluent | PBS containing 1% BSA and 0.3% TritonX-100 |
| PBST | PBS containing 0.1% Tween20 |
| Primary antibody 1 | Anti-Fibronectin1 mouse monoclonal antibody |
| Primary antibody 2 | Anti-Vinculin rabbit monoclonal antibody |
| Secondary antibody 1 | Alexa Fluor 555-conjugated anti-mouse IgG antibody |
| Secondary antibody 2 | Alexa Fluor 647-conjugated anti-rabbit IgG antibody |
