# Supplementary figures and images for: Enhancing cell adhesive and antibacterial activities of glass-fibre-reinforced polyetherketoneketone through Mg and Ag PIII
Source: Regen Biomater. 2023 Jul 12;10:rbad066. doi: 10.1093/rb/rbad066 (PMC10363026; doi:10.1093/rb/rbad066)

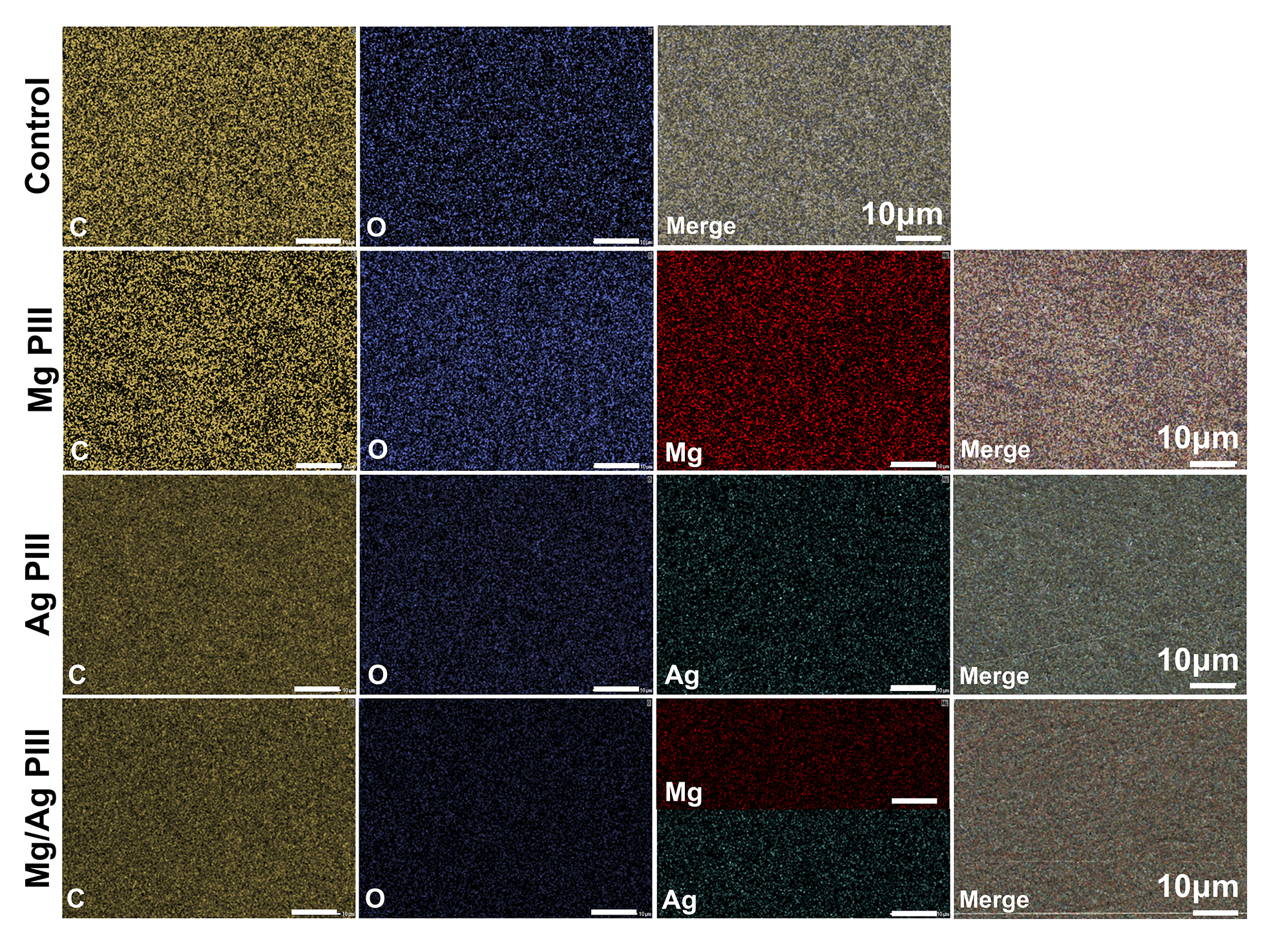

Supplement: rbad066_Supplementary_Data [file rbad066_supplementary_data.zip › Figure S1.jpg]
